# S51. MOTIVATIONAL ENHANCEMENT IMPROVES TREATMENT OUTCOMES OF MOBILE-BASED COGNITIVE REMEDIATION IN INDIVIDUALS WITH SCHIZOPHRENIA

**DOI:** 10.1093/schbul/sby018.838

**Published:** 2018-04-01

**Authors:** Eun Jin Kim, YongChun Bahk, Kee-Hong Choi

**Affiliations:** Korea University

## Abstract

**Background:**

Cognitive impairment is a core feature of schizophrenia, which limits functions of individuals with schizophrenia and negatively influences their quality of life (Green, 1993; Green et al, 2000; Heaton et al., 2001; Heinrichs, 1998). While pharmacological treatment is known to have a limited effect on impaired cognition in schizophrenia (Marder, 2006; Rund and Borg, 1999; Elie et al., 2010), a majority of literature has concluded that cognitive remediation(CR) produces small to moderate improvements (McGurk et al., 2007; Wykes et al., 2011). As the smartphone user population continues to increase, the effectiveness of CR based on mobile devices have started to be studied. While CR is effective in improving cognitive deficit, treatment adherence and engagement of participants in the real world setting is known to be poor compared to laboratory setting. Thus, in the current randomized controlled study, we aimed to investigate whether motivational intervention would enhance motivation, treatment adherence and neurocognitive function of individuals with schizophrenia.

**Methods:**

All subjects participated in a group-based CR using mobile application (mCR) twice a week for five weeks, and were given opportunity to practice voluntarily outside the treatment sessions. While CR only group participated in usual CR with Q&A sessions, experimental group participated CR sessions integrated with motivational intervention. For motivational enhancement (ME), we employed principles (e.g., goal setting, linking of CR with life goals, etc) of the bridging group (Medalia, Revheim, & Herlands, 2009) along with key aspects of motivational interviewing (e.g., open end questions, affirmation, reflect, and summary). We hypothesized that compared to CR only group, CR+ME group would show higher levels of intrinsic motivation, attendance rate and extra voluntary training hours, and greater improvement in cognitive functions.

**Results:**

We are undergoing the current project, and a total of 14 participants were randomly assigned to either CR+ME (n=8) or CR only (n=5). Among 14 participants, two participants dropped out (n=1 experimental group and n=1 control group).

Independent sample t-test were used to compare scores of demographics and clinical characteristics between groups, and no differences were found except for the PANSS excitement subscale (t = 2.91, P < .05) at the time of pre-treatment. Due to a small sample, we conducted paired sample t-tests to examine whether there was a significant difference between the pre and post-test for two groups, respectively. The paired t-test revealed improvements in coding, TMT-B, logical memory I and K-AVLT immediate recall performances of CR+ME (t=-2.92, p < .01; t=-3.65, p < .05; t=-3.20, p < .05; t=-2.89, p < .05), but not CR only. In addition, there were pre and post-treatment differences in motivation variables (MSQ) for CR+ME. Comparing task related motivational level of first session to the final 10th session, CR+ME showed increased identified regulation(IR) score of MSQ and decreased external regulation(ER) score (IR=22.3(3.2), 23.5(3.7); ER= 10.67 (3.88), 6.67 (3.08)).

**Discussion:**

We conclude that ME is promising to further enhance neurocognitive and motivational outcomes of mCR. The data collection process is expected to be completed in late January 2018, and the results will be accordingly updated by the time of presentation at SIRS 2018. Limitations and future directions will be discussed.

